# Enhancing the performance of porous silicon biosensors: the interplay of nanostructure design and microfluidic integration

**DOI:** 10.1038/s41378-024-00738-w

**Published:** 2024-07-17

**Authors:** Kayan Awawdeh, Marc A. Buttkewitz, Janina Bahnemann, Ester Segal

**Affiliations:** 1 Faculty of Biotechnology and Food Engineering, Technion—Israel Institute of Technology, 320003 Haifa, Israel; 2 Institute of Technical Chemistry, Leibniz Universität Hannover, 30167 Hannover, Germany; 3 Institute of Physics, University of Augsburg, 86159 Augsburg, Germany; 4 Centre for Advanced Analytics and Predictive Sciences (CAAPS), University of Augsburg, 86159 Augsburg, Germany

**Keywords:** Biosensors, Structural properties, Microfluidics

## Abstract

This work presents the development and design of aptasensor employing porous silicon (PSi) Fabry‒Pérot thin films that are suitable for use as optical transducers for the detection of lactoferrin (LF), which is a protein biomarker secreted at elevated levels during gastrointestinal (GI) inflammatory disorders such as inflammatory bowel disease and chronic pancreatitis. To overcome the primary limitation associated with PSi biosensors—namely, their relatively poor sensitivity due to issues related to complex mass transfer phenomena and reaction kinetics—we employed two strategic approaches: First, we sought to optimize the porous nanostructure with respect to factors including layer thickness, pore diameter, and capture probe density. Second, we leveraged convection properties by integrating the resulting biosensor into a 3D-printed microfluidic system that also had one of two different micromixer architectures (i.e., staggered herringbone micromixers or microimpellers) embedded. We demonstrated that tailoring the PSi aptasensor significantly improved its performance, achieving a limit of detection (LOD) of 50 nM—which is >1 order of magnitude lower than that achieved using previously-developed biosensors of this type. Moreover, integration into microfluidic systems that incorporated passive and active micromixers further enhanced the aptasensor’s sensitivity, achieving an additional reduction in the LOD by yet another order of magnitude. These advancements demonstrate the potential of combining PSi-based optical transducers with microfluidic technology to create sensitive label-free biosensing platforms for the detection of GI inflammatory biomarkers.

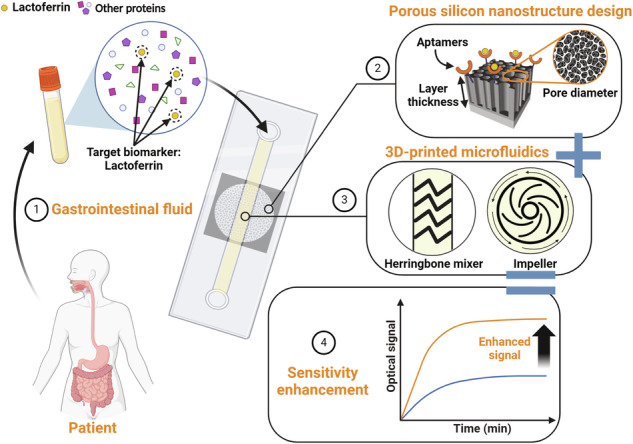

## Introduction

Surface-based transducers have been extensively utilized to develop bioanalytical sensors. The performance of these sensors relies on the interconnected effects of the mass transfer flux of the analyte from the solution toward the surface, as well as the reaction kinetics that arise between the analyte and the immobilized recognition element^1–3^. As binding of the target occurs, however, its concentration in the vicinity of the biosensor surface is rapidly depleted—resulting in the creation of a diffusion boundary layer that extends into the bulk solution^1,4^ and eventually negatively affects the biosensor’s performance. This complex interplay between hindered mass transfer and the reaction kinetics becomes even more pronounced in porous-based biosensors^4–9^. While such transducers have demonstrated tremendous potential in numerous diagnostic applications (owing to numerous advantageous structural features which include a substantial specific surface area, reactivity, and tunable structural properties such as pore diameter and shape)^10^, the mass transport phenomena of the analyte continue to pose persistent challenges^11–14^. Recently, we developed a comprehensive model that describes the mass transfer phenomena in porous transducers (with an emphasis on porous silicon (PSi) transducers) and considers factors such as the target diffusion within the bulk solution, hindered diffusion, and target–bioreceptor binding kinetics^4,15,16^. Among porous materials, PSi-based biosensors are generally considered to be particularly promising optical transducers because they facilitate label-free and real-time detection of various targets. However, the mass transfer phenomena noted above has thus far restricted them to the micromolar range—which is a serious limitation since many protein disease biomarkers exist at extremely low concentrations that only fall within the subnanomolar range. Consequently, significant efforts are currently being directed toward improving the performance of these biosensors by designing flow-through platforms^17–19^, integrating signal amplification methods (such as the use of secondary capture probes^20^ or labeled molecules^21–23^), and employing signal processing strategies (such as interferogram average over wavelength reflectance spectra^24^ or Morlet Wavelet convolution^25^). Importantly, previous works have emphasized that tuning the various characteristics of a porous biosensor (such as its porous layer thickness, pore diameter, and capture probe density) can profoundly affect the target capture rate and enhance its overall performance^4,14,16,19,26–28^.

Another practical approach to minimizing mass transfer limitations in both planar and porous biosensors is to employ microfluidic systems, in which the diffusion path length between the analyte in the bulk solution and the biosensor surface is decreased to facilitate faster mass transfer and enhance the sensitivity compared to conventional setups^29–31^. In previous studies, PSi-based optical biosensors have been integrated within plain microchannels that are typically made of polydimethylsiloxane (PDMS), resulting in a pronounced enhancement in the performance of these biosensors—an outcome which can be primarily attributed to a reduction in the diffusion length of the analyte to the porous surface^5,32–34^. While the vast majority of reported microfluidic devices employ soft lithography techniques and PDMS^35^, their widespread adoption continues to be hindered by the complex fabrication processes and scalability challenges that plague these technologies more generally. Conversely, three-dimensional (3D)-printed microfluidic devices offer a promising alternative due to their flexible design, simplicity, efficiency, and versatility^29,30,36,37^. As a result of these advantages, novel and sophisticated microfluidic designs with versatile geometries and components can be readily designed and easily fabricated, and mixing components embedded in such microfluidic systems can also allow for further flux enhancement and mass transfer acceleration. These mixing components can be classified into two camps: active and passive mixers^38–40^. Active mixers, such as those involving mechanical stirring, rely on external energy sources that can physically agitate the liquid in a microchannel, offering controllable and efficient mixing of the analyte solution while simultaneously delivering fresh analyte to the biosensor surface^38,39^. In contrast, passive mixers operate without external actuators, and instead mostly rely on hydrodynamic manipulation of the fluids^38^. Different passive mixers with different geometries, including staggered herringbone micromixers (SHMs)^41^ and Y-shaped geometry mixers^42^, have been proposed in the literature. These mixers generate helical flows, thereby increasing the chance of target–surface interactions and minimizing depletion at the sensor surface^43^.

In this work, we present a new aptasensor that utilizes PSi Fabry‒Pérot thin films as optical transducers for the detection of lactoferrin (LF), which is a protein released during inflammation. Lactoferrin is a critical biomarker for diagnosing and monitoring gastrointestinal (GI) inflammatory disorders, including inflammatory bowel disease and chronic pancreatitis^44,45^. Our aim was to design a biosensor capable of functioning in GI fluid—which is a complex biofluid that contains a high concentration of many different biomolecules. Critically, such a biosensor must be sufficiently sensitive to enable detection of clinically relevant concentrations (>10 nM^46–50^) of the target biomarkers of interest. To enhance the performance of PSi, we employed two approaches. The first approach focused on rational design of the porous nanostructure, and specifically we investigated the impact of PSi nanostructure properties—such as the porous layer thickness, pore diameter, and capture probe density—on the binding kinetics within the pores. Based on our mass transfer model^4^, we hypothesized that decreasing the porous layer thickness, increasing the pore diameter, and achieving an optimal probe surface density would significantly enhance the target diffusion and capture within the pores. The second approach involved integrating the PSi biosensor into 3D-printed microfluidic systems with different micromixer architectures (i.e., SHM structures or microimpellers) embedded. The role of convection in such systems was a primary focus of this investigation, since effective mixing is expected to minimize the formation of a depletion region near the pore entry, which limits the biosensor sensitivity^1^.

## Materials and methods

### Materials

Si wafers (highly doped p-type, <100>-oriented, with a characteristic resistivity of ~0.95 mΩ cm) were obtained from Sil’tronix Silicon Technologies (Archamps, France). Absolute ethanol was provided by Bio-Lab Ltd. (Jerusalem, Israel). Aqueous hydrofluoric acid (HF) 48%, N-hydroxysuccinimide (NHS), N-ethyldiisopropylamine (EDIPA), (3-aminopropyl)triethoxysilane (APTES), methoxypolyethylene glycol amine 750 Da (PEG), succinic anhydride, acetonitrile (ACN), N-(3-dimethylaminopropyl)-N′-ethylcarbodiimide hydrochloride (EDC), 2-(n-morpholino)ethanesulfonic acid (MES), MES sodium salt, Tris base, lactoferrin from bovine milk, trypsin, bovine serum albumin (BSA), and buffer salts were supplied by Sigma‒Aldrich Chemicals (Rehovot, Israel). All buffer solutions were prepared using Milli-Q water (ddH_2_O, 18.2 MΩ cm). The 3’-amino-modified anti-LF aptamer Lac 9-2 ^51^ (5’- CA GGC AGG ACA CCG TAA CCG GTG CAT CTA TGG CTA CTA GCT CTT CCT GCC TAT TTT TTT TTT-3’) was purchased from Integrated DNA Technologies (Coralville, USA). Phosphate-buffered saline (PBS, pH 7.4) composed of NaCl (137 mM), Na_2_HPO_4_ (10 mM), KCl (2.7 mM), and KH_2_PO_4_ (2 mM) was used. Selection buffer (SB, pH 7.4) was prepared by dissolving MgCl in PBS (pH 7.4) to a concentration of 1 mM. The MES buffer (pH 6) consisted of MES (0.27 M) and MES sodium salt (0.23 M). The printing material (AR-M2) and support material (AR-S1) for 3D printing of the microfluidic devices were purchased from Keyence Corporation (Osaka, Japan). Medical Tape 9877 was purchased from 3M (St. Paul, USA). GI fluids were supplied by Given Imaging Ltd. The GI fluids were obtained from domestic pigs, *Sus scrofa domesticus* (large White mixed with Landrace, aged 5.5 months and weighing 90 kg), from the Lahav Research Institute according to ethical approval IL-17-8-290 (The Israel National Ethics Committee).

### Construction of the PSi aptasensor

#### Silicon anodization

Anodization was carried out in a solution of HF and ethanol at a ratio of 3:1 (v/v), as we have previously described^52,53^. The anodization time and current density were varied to yield PSi films with different pore sizes and layer thicknesses. Films with 50 nm-wide pores were fabricated at a current density of 375 mA cm^−2^ with an etching time that varied between 12 and 30 s; for films characterized by 80-nm-diameter pores, the conditions were 75 mA cm^−2^ for 70 s or 120 s. Subsequently, 1 h of thermal oxidation (800 °C) in a tube furnace (Lindberg/Blue M 1200 °C Split-Hinge, Thermo Scientific, USA) was performed.

#### PSi functionalization

Amino-terminated anti-LF aptamers (Lac 9-2^51^) were immobilized onto the porous nanostructure by carbodiimide coupling chemistry, as previously described^32,54^. Initially, the oxidized PSi films were immersed in APTES (1% v/v) and EDIPA (1% v/v) solution in ddH_2_O for 1 h and then extensively rinsed with ddH_2_O and ethanol. Subsequently, annealing was performed at 100 °C for 15 min. Once cooled to room temperature, the amine-terminated PSi was incubated with succinic anhydride (10 mg ml^−1^) and EDIPA (2% v/v) in acetonitrile for 3 h. The PSi films were then thoroughly washed with acetonitrile and ddH_2_O. Next, the films were reacted for 1 h with NHS (5 mg ml^−1^) and EDC (10 mg ml^−1^) dissolved in MES buffer, followed by thorough rinsing with MES buffer. Subsequently, the samples were incubated with a solution of amino-terminated aptamers in PBS for 1 h and washed with Tris buffer (50 mM, pH 7.4) to deactivate the remaining NHS and EDC moieties. Alternatively, these groups were deactivated by conjugation of 1 mg ml^−1^ Me-PEG-NH_2_ (750 Da) to the aptamer-modified PSi via incubation for 1 h, followed by washing with PBS for 15 min and Tris buffer.

For functionalization of PSi integrated within the microfluidic devices, the NHS/EDC activation and subsequent steps were carried out within the microchannels. An NHS/EDC solution in MES (as described above) was then introduced at a rate of 30 µl min^−1^ for 30 min. Afterward, a solution of the aptamer in PBS (10 µM, 250 µl) was introduced under similar flow conditions and incubated for 1 h. The same procedure was used for immobilization of PEG-NH_2_ using a solution of Me-PEG-NH_2_ in PBS at a concentration of 1 mg ml^−1^. Finally, Tris buffer was introduced to the microchannels for 15 min at a flow rate of 30 μl min^−1^.

### Characterization of the PSi aptasensor

#### Scanning electron microscopy (SEM)

The nanostructure of PSi, in terms of the pore diameter and porous layer thickness, was characterized via high-resolution SEM (Carl Zeiss Ultra Plus) at an acceleration voltage of 1 keV.

#### Fourier transform infrared (FTIR) spectroscopy

The immobilization of the aptamers was characterized using attenuated total reflectance FTIR spectroscopy (Thermo 6700 FTIR equipped with a Smart iTR diamond ATR).

### Design and fabrication of 3D-printed microfluidic devices

The 3D models were designed via computer-aided design (CAD) software using SolidWorks 2022 (Dassault Systèmes SolidWorks Corp, Waltham, MA, USA). The models were saved as an .STL file and then printed with a Agilista-3200W high-resolution 3D printer (Keyence Corporation, Osaka, Japan) using AR-M2 as the printing material and AR-S1 as the support material. The devices were printed with X, Y, and Z resolutions of 40, 64, and 20 µm, respectively. The devices were then removed from the printing platform and submerged in an ultrasonic bath (Elma Elmasonic S30, Elma, Schmidbauer GmbH, Singen, Germany) with ddH_2_O and detergent at 60 °C for 15 min to remove the support material. The interior channels were washed with an aqueous detergent solution, and this cleaning process was repeated at least three times. Finally, the devices were rinsed with ddH_2_O and dried at 70 °C for 1 h.

### Flow velocity profile simulations

The simulations were performed using SolidWorks Flow Simulation (2022). Internal analysis type was chosen with physical features of fluid flow and rotation. Water was used as the fluid at room temperature under a pressure of 1 atm. The boundary conditions were set to a fully developed flow perpendicular to the channel, where the flow rate was set to 30 μl min^−1^ and the outlet pressure was set to 1 atm. An initial mesh level of 3 was used. The particle study consisted of 100 traceable particles (8 nm in diameter to mimic the size of LF^55^) dispersed in water. The constraints were set to a length of 4 cm, a duration of 3600 s, and 5000 iterations. For the wall conditions, we used reflection throughout the microchannel length and adsorption in the predefined biosensing area (2.6 mm^2^) to count the particles coming into contact with the surface.

### Integration of PSi with microfluidics

The PSi films were integrated with 3D-printed microfluidic devices by utilizing double-sided adhesive tape (Medical Tape 9877) with a thickness of 110 µm. The tape was cut into 1.8 mm squares, with an additional cutout tailored to the microchannel/mixing chamber. Subsequently, the tape was affixed to the bottom part of the 3D-printed device, thereby ensuring alignment of the cutout with the corresponding cavity in the device. Following this step, a PSi chip was carefully positioned on the tape, orienting the etched structure with the 3D-printed device. To ensure an optimal bonding interface, 3D-printed parts on the top and bottom of the assembled device were fitted and aligned, and a pressure of 25 kPa was applied at 70 °C for 1 h.

### Biosensing experiments

Reflective interferometric Fourier transform spectroscopy (RIFTS) was used to detect real-time refractive index changes occurring within the PSi film. Reflectance spectra were collected using a charge-coupled device (CCD) USB 4000 spectrometer (Ocean Optics, USA) connected to a bifurcated fiber-optic cable and a collimator. The aptasensor was fixed in a custom-made flow cell or integrated within the microfluidics devices, and a tungsten light source with a spot size of ~1 mm^2^ was focused on the center of the PSi sample. Reflectivity spectra were recorded every 15 s throughout the experiments and analyzed by applying fast Fourier transformation (FFT) in the wavelength range of 450–900 nm. The resulting FFT spectrum exhibits a single peak, and the position of this peak corresponds to the value of 2*nL*—termed the effective optical thickness (EOT)—where *n* refers to the average refractive index and *L* refers to the PSi thickness. The biosensing experiments were performed either at room temperature when working only with buffer solutions or at 37 °C when working with GI fluids. For all experiments, the aptasensor was first washed with elution buffer (2 M NaCl in ddH_2_O) for 15 min to unfold the aptamers, followed by incubation in SB for 30 min, during which a stable reflectance baseline was achieved. Next, the protein solution in SB was introduced for 1 h, followed by removal of the solution and a washing step (SB for 30 min) to remove the unbound proteins. For experiments conducted with GI fluids, the aptasensor was exposed to neat GI fluid for at least 30 min to acquire a stable baseline, followed by the exposure of the biosensor to spiked GI fluids for 1 h and a washing step with GI fluids for 30 min. For biosensing experiments carried out inside the microchannels, the different solutions were introduced at a flow rate of 30 μl min^−1^.

The results are presented as the relative ΔEOT:

$$\frac{\Delta {{\rm{EOT}}}_{{{t}}}}{{{\rm{EOT}}}_{0}}=\frac{{{\rm{EOT}}}_{{{t}}}-{{\rm{EOT}}}_{0}}{{{\rm{EOT}}}_{0}}$$

where EOT_0_ is the average EOT value during baseline acquisition in SB, and EOT_*t*_ is the average EOT value attained at equilibrium following the removal of the protein after incubation and subsequent washes. The average slope of the real-time EOT curves collected 10 min after protein introduction was calculated.

The signal-to-noise ratio (SNR) was determined by calculating the ratio of the relative EOT signal to the standard deviation (*σ*) of the signal prior to the introduction of protein solutions in SB. The limit of detection (LOD) was set as 3*σ* the noise level. Analogous to the LOD, the limit of quantification (LOQ) was calculated as 10*σ*. The relative standard deviation (%RSD) was calculated by dividing *σ* by the average relative EOT change.

The dissociation constant (*K*_*D*_) was determined via nonlinear regression of the collected data using a specific binding model with a Hill slope according to the following equation:

$$Y=\frac{{B}_{\max }\cdot X}{({K}_{D}+X)}$$

where *B*_max_ represents the concentration at which the maximum biosensor response is reached^56^. GraphPad Prism software was used for fitting.

### Statistical analysis

All experiments were repeated at least three times. The data are presented as the mean of *n* ≥ 3 along with the standard deviation of the mean. For statistical analysis, an unpaired *t*-test with a two-tailed distribution and unequal variance was used. A *p* value of <0.05 was considered to indicate a statistically significant difference between groups.

## Results and discussion

### PSi aptasensor construction

**Fig. 1:**
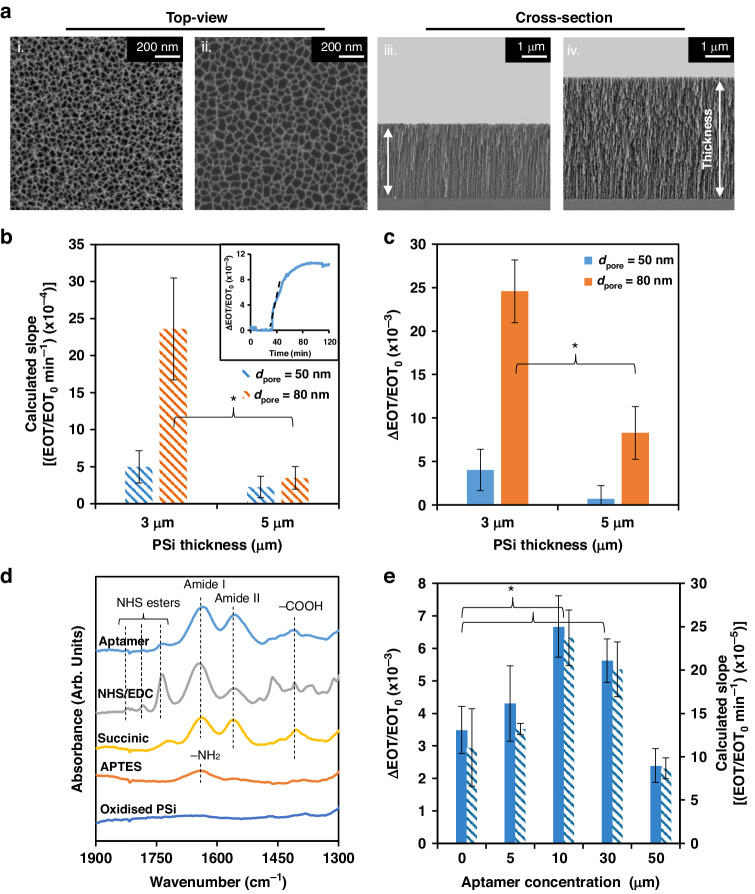
PSi nanostructure properties and Lac 9-2 immobilization. **a** Electron micrographs of oxidized PSi films: (i) top view of the film with an average diameter of 50 or (ii) 80 nm and (iii) cross-section of the film with a porous layer thickness of 3 or (ii) 5 µm. **b** Effect of the PSi layer thickness for different pore diameters on the calculated infiltration slope and (**c**) the net EOT changes upon exposure of the aptasensor to 8 μM LF (the inset presents a characteristic real-time EOT curve and the calculated infiltration slope). *d*_pore_ is the average pore diameter. **d** ATR-FTIR spectra of the PSi film following the chemical modification steps for aptamer immobilization. **e** Effect of the capture probe concentration on the biosensing performance. (* indicates statistical significance, *t-*test, *n* ≥ 3, *p* < 0.05)

Several structural properties of the PSi nanostructure—such as the pore diameter and thickness of the porous layer—can be readily tuned during the fabrication process by varying the etching conditions, current density, and time. These characteristics have a strong impact on the binding rate, due to their effect of hindering diffusion of the target molecule within the pores (since this is the main limiting factor of PSi-based biosensors)^4,32,57^. Accordingly, we studied the effect of the porous layer dimensions (i.e., pore diameter and thickness) on LF protein (~87 kDa) infiltration into the nanostructure. To facilitate effective infiltration of a molecule into the porous layer, a critical correlation between the size of the molecule and the diameter of the pores has been suggested in which the pore diameter must be at least five times larger than the molecule size^4,57,58^. Accordingly, since the size of the LF molecule in question was ~8 nm^55^, the etching conditions were adjusted to yield pores with a pore opening larger than 40 nm. We fabricated films with pores of approximately 50 nm or 80 nm, and top-view HRSEM images of the resulting layers revealed irregularly shaped pores with the desired average diameter (see Fig. 1a-i, ii, respectively). Note that the pores were intentionally designed with diameters larger than 40 nm, since the effective pore diameter decreases following aptamer immobilization within the nanostructure^4,57^. This design ensured that even after the aptamers are conjugated, the pores still remained large enough to facilitate the diffusion and capture of lactoferrin molecules. The porous film thickness varied between 3 and 5 µm, as shown in the respective cross-section electron micrographs in Fig. 1a(iii-iv); as our previous simulation work indicated, a reduction in the porous layer thickness is essential for minimizing hindered diffusion effects while maintaining the integrity of the EOT signal^4,52,59,60^. This signal is derived from the raw reflectance spectrum, which exhibits a characteristic Fabry–Perot interference pattern (see Table S1 Supporting Information). LF solution was introduced into the different porous layers following their oxidation, and changes in the EOT over time were collected. Figure 1b, c presents a characteristic real-time EOT curve (inset), and summarize the relative EOT attained after 1 h and the calculated slope values for the different nanostructures. The pore diameter was found to have the most pronounced effect on protein infiltration in terms of both the infiltration rate and the amount of protein that accumulated within the pores. In the case of 50 nm pores, no significant difference in the calculated infiltration slopes was observed across the different porous layer thicknesses, and only minor EOT changes were detected, indicating poor LF infiltration into the nanostructure. By contrast, when the average pore diameter was increased to 80 nm, both the infiltration rate and the net EOT also increased by >5-fold. A thinner layer (3 µm) allowed both higher infiltration rate (7-fold) and EOT change (3-fold), which can be attributed to the shorter diffusion length and higher concentration gradient—which is the driving force for diffusion. Regarding thicker layers, a rapid depletion zone at the pore entrance was observed to form, likely owing to the fast uptake of the target^61–63^. These results align well with our previous theoretical simulation results, which demonstrated that for capture probes with high-affinity interactions (*k*_on_ of 10^5^ M^−1^ s^−1^ and *k*_off_ of 10^−4^ s^−1^) similar to the characteristics of the Lac 9-2 aptamer, the effect of the nanostructure is crucial and more pronounced than that for lower-affinity interactions^4^. Based on these results, the pore diameter and layer thickness were selected to facilitate high LF diffusion flux and pore infiltration, both of which are critical factors in the performance of porous-based biosensors^4,63^.

As a capture probe, we utilized the Lac 9-2 aptamer, which is composed of a 52-base-long binding region and was specifically selected to target the LF protein^51^; accordingly, this aptamer exhibits high affinity for LF, with a *K*_D_ of 1.121 nM. A 10-thymine-base spacer at the 3′-terminus of the aptamer was used to increase the distance between the binding region and the solid PSi surface^64,65^. The amine-terminated aptamer was conjugated to the thermally oxidized PSi via NHS/EDC coupling chemistry^66^, which is presented in Fig. S1 (Supporting Information). The conjugation process was monitored using ATR-FTIR spectroscopy, and each step was evident in the corresponding spectrum (Fig. 1d). After amino-silanization, a peak at 1640 cm^−1^ was observed, which could be ascribed to the bending of the primary amines^32,67^. Carboxylation of the surface with succinic anhydride introduced two new prominent peaks at 1557 and 1637 cm^−1^, corresponding to amide II and amide I bonds, respectively. Additionally, the new peak at 1406 cm^−1^ was associated with carboxylic acid groups^32,67^. After activation with EDC and NHS, the spectrum exhibited three peaks at 1736, 1785, and 1820 cm^−1^, which are characteristic of NHS esters on the surface^32,67,68^. The last peak diminished following the introduction of the amine-terminated aptamer, while the amide I and II bond peaks actually intensified—suggesting successful immobilization of the aptamers. To deactivate the reactive NHS and EDC groups remaining on the surface, blocking with Tris was performed (as illustrated in Fig. S1). Tris, which is a small hydrophilic molecule, is commonly immobilized on various surfaces as an antibiofouling agent and on biosensors to minimize nonspecific adsorption^32,69–73^.

The spatial surface density of the aptamers immobilized within the pores was optimized by studying the effect of increasing aptamer concentration between 5 and 50 µM on the biosensing performance. Figure 1e summarizes the respective aptasensor response in terms of the attained relative EOT values and the calculated slope following the introduction of LF solution (1 µM). The highest response was obtained when a Lac 9-2 concentration of 10 µM was used, indicating that at this aptamer concentration, an optimal surface density was achieved. A further increase in the aptamer concentration impaired the aptasensor performance, which can be attributed to steric hindrance effects^4–6,74–77^.

### Aptasensor selectivity and sensitivity

**Fig. 2:**
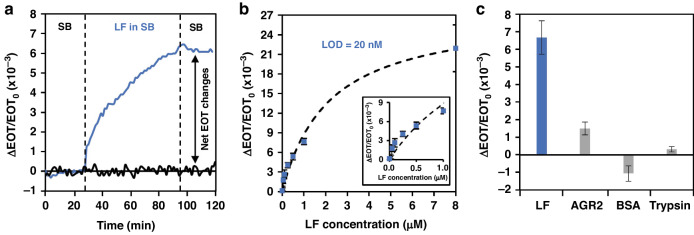
Performance of the aptasensor in buffer. **a** Real-time relative EOT changes upon introduction of a 1 µM LF solution (blue trace) or neat SB (black trace). **b** Net relative EOT changes upon exposure of the aptasensor to LF at different concentrations (0–8 µM) (the inset presents the net EOT changes for low concentrations (0–1 µM)). **c** Net relative EOT changes upon exposure of the aptasensor to LF and different nontarget protein solutions (trypsin, BSA, and AGR2) at a similar concentration of 90 μg ml^−1^, which is equivalent to 1 µM for LF

Figure 2a presents the real-time relative EOT changes of the PSi aptasensor that were obtained upon the introduction of the target protein (LF at 1 μM). Initially, SB was introduced to allow for proper folding of the aptamer and acquisition of an initial EOT baseline. LF introduction induced a gradual increase in the EOT signal due to the infiltration of the protein into the porous layer and its subsequent binding to the tethered aptamers. After a 1-h incubation period, SB was introduced to remove unbound and nonspecifically adsorbed LF molecules. Nevertheless, a negligible decrease in the EOT was observed, demonstrating strong binding between the target protein and the tethered aptamer.

The aptasensor performance was investigated by introducing LF at various concentrations between 0 and 8 µM (Fig. 2b). The lowest measured concentration was 50 nM, with a relative EOT increase of (1.8 ± 0.5) × 10^−3^ and an SNR of 2.3 ± 0.7. The curve was fitted with a sigmoidal curve (*R*^2^ = 0.9816), and accordingly *K*_D_ was estimated to be 2.1 ± 0.9 µM. This value is higher than the reported value for the selection of the anti-LF aptamer (in the nanomolar range) determined by Ag nanoparticle-enhanced surface plasmon resonance imaging^51^. The aptasensor showed high sensitivity to LF, with a calculated LOD of 20 nM, which is >1 order of magnitude lower than previously reported LOD values (0.21–2.7 μM)^32,71,72^ for PSi-based aptasensors in which a thicker porous layer of ~5 μm and aptamers with higher dissociation constants (0.013 to 4.6 × 10^−6^ M) were used. This illustrates the profound effect of the PSi structural properties and binding affinity on the biosensor sensitivity.

The selectivity of the aptasensor was investigated by exposing it to biologically relevant nontarget proteins (including anterior gradient homolog-2 (AGR2), bovine serum albumin (BSA), and trypsin) and monitoring its response, as shown in Fig. 2c. These proteins were found to induce only negligible changes in the relative EOT signal, demonstrating the high selectivity of the aptasensor for LF.

**Fig. 3:**
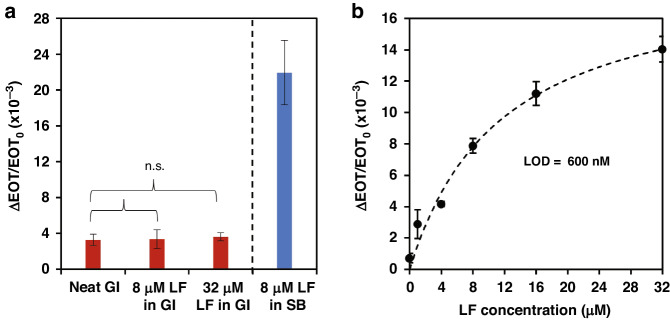
Aptasensor performance in GI fluids. **a** Net relative EOT changes for neat GI, GI with 8 µM and 32 µM LF, and buffer with 8 µM LF. **b** Net relative EOT changes upon exposure of the aptasensor passivated with PEG to GI spiked with LF at different concentrations (0–32 µM). (*t-*test, *n* ≥ 3, *p* < 0.05, n.s. indicates no statistical significance)

After establishing the excellent performance of the aptasensor in buffer solutions, we proceeded to examine its ability to perform within GI fluids (complex biofluids containing a high concentration of many different nontarget biomolecules)^71,78,79^. GI fluids collected from domestic pigs were used to mimic the conditions of the human GI tract^80,81^, and the total protein content in these samples was measured as ~10 mg ml^−1^. Figure 3a shows the response of the aptasensor to neat GI and LF-spiked GI fluids (LF concentrations of 8 and 32 µM). The aptasensor was found to be insensitive to LF; regardless of the LF concentration, the biosensor response was similar (i.e., displaying statistically insignificant relative EOT changes) to that for neat GI. Note that the pH and ionic strength of the GI fluids differed from the conditions used for the Lac 9-2 aptamer selection process^51^, which might have negatively affected the binding affinity of the tethered Lac 9-2 to LF^82,83^. Nevertheless, the aptasensor insensitivity also suggested that the PSi surface may have been saturated with physiosorbed nontarget biomolecules; regarding this point, note that the EOT signal in Fig. 3a remained unchanged (no statistical difference) throughout the wide range of protein loadings. Thus, Tris passivation of the aptasensor may prove ineffective in preventing biofouling in complex GI fluid. In our recent work^71^, covalent conjugation of polyethylene glycol (PEG) molecules to PSi nanostructures was demonstrated to provide effective biofouling resistance in complex biofluids while preserving the target binding activity of the tethered aptamers. Therefore, amine-modified PEG (750 Da) was immobilized on the biosensor surface following Lac 9-2 conjugation (as illustrated in Fig. S1, Supporting Information). The resulting aptasensor performance in buffer was examined and was found to be comparable to that of the Tris-passivated biosensor (see Fig. S2, Supporting Information, for comparison). Importantly, upon exposure to GI spiked with LF at various concentrations, a characteristic binding curve was observed (Fig. 3b) with a calculated apparent dissociation constant of 11.8 ± 1.9 nM and a LOD of 600 nM. While PEG conjugation allows LF detection in a protein-rich medium, the aptasensor sensitivity was >1 order of magnitude lower than that achieved in buffer and is irrelevant for clinical application. Therefore, we next aimed to integrate the aptasensor with microfluidics to improve its limited sensitivity.

### Mass transfer acceleration

**Fig. 4:**
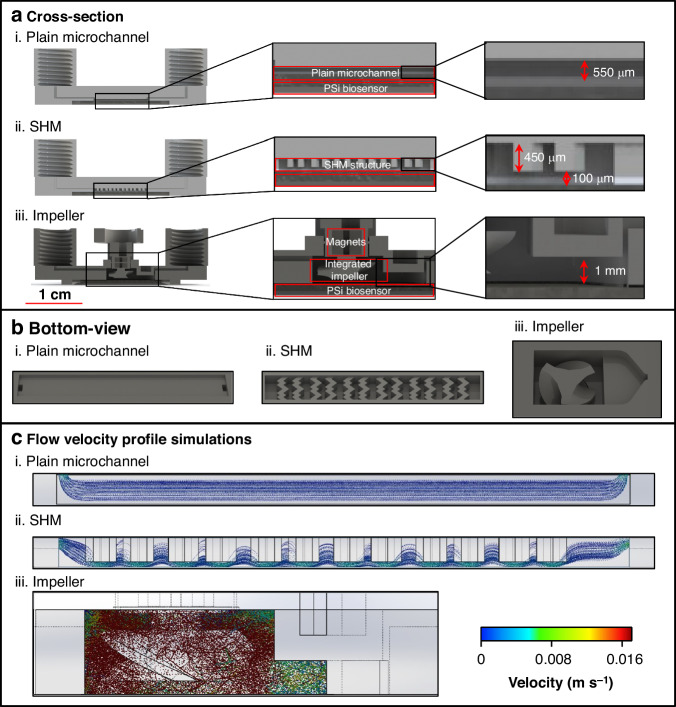
3D-printed microfluidics design and flow simulations. **a** Cross-sectional and (**b**) bottom views and (**c**) simulated flow velocity profiles of the different microfluidic designs: (i) Plain microchannel with a width of 1.6 mm and a height of 550 μm; (ii) SHM-structured microchannel (dimensions similar to the plain microchannel) with a herringbone structure (450 μm in height); (iii) Microimpeller system containing a mixing chamber to accommodate the impeller and a measurement zone with a height of 1 mm. All the simulations were conducted using SolidWorks. The colors of the particles correspond to their velocities, with blue indicating low values and red indicating high values

We propose two different 3D-printed microfluidic designs incorporating micromixers, both of which aim to enhance the target flux to the porous layer. These designs consist of a staggered herringbone mixer (SHM) microstructure and an impeller, which are used to achieve passive and active mixing, respectively (see Fig. 4a, b). The SHM-structured microchannel consists of two repeating ridge patterns, with alternation between two identical ridges of each pattern along the microchannel (see Figs. 4a-ii and 4b-ii). The change in the ridge orientation induces an exchange of rotation centers, thereby augmenting mixing by creating chaotic advection and disrupting the formation of boundary layers^40,84^. This structure has already been shown to induce efficient passive micromixing in various biosensing formats^40,41,43,84,85^. For example, a similar PDMS-based SHM structure coupled to an electrochemical aptasensor was shown to exhibit an LOD of 0.2 pM in buffer^85^.

Our primary challenge in designing microfluidic structures suitable for production via 3D printing stems from the limitations imposed by the current printer resolutions and the minimal printable feature size—which is currently larger than that of PDMS-based microfluidics fabricated by soft lithography^86^. Consequently, we selected a ridge height of 450 µm (which is needed for optimal reproducible printing) within a microchannel of 550 µm (see Fig. 4a, b(ii)). A plain microchannel with similar dimensions was employed for comparison, as illustrated in Fig. 4a, b(i).

The second mixing configuration consisted of a miniaturized monolithic 3-blade impeller accommodated within the mixing chamber above the biosensor surface, as depicted in Fig. 4a, b(iii) (for the detailed geometry and dimensions, see Fig. S3, Supporting Information). The impeller is driven by an external motor connected via disc-shaped magnets mounted atop the impeller. This microimpeller design, which is studied here for the first time, was intended to enhance the mixing of the target by minimizing the creation of a depletion zone at the pore entrance and enhancing the reaction kinetics^38,40,87^. Moreover, the implementation of the microimpeller was also intended to provide precise control over the mixing parameters in terms of both the stirring speed and duration^38,40,87^. This, in turn, was expected to yield improved mixing efficiency^88^ compared to passive structures, particularly the SHM.

The micromixer design process was supported by computational fluid dynamics (CFD) simulations using SolidWorks and incorporating particle tracing (using 8-nm spherical particles). Note that diffusion was not considered in these simulations. The results are presented in Fig. 4c, where the target particle trajectories are depicted along with their respective velocities. The interaction chance between the target and the aptasensor surface was calculated based on these simulation results. The SHM induced interchanging flowlines, breaking the parallel flow in the plain microchannel, with accelerated particle movement beneath the ridges (Fig. 4c-ii). This resulted in a significant increase in the interaction chance of the target with the surface of up to 14%, whereas for the plain microchannel, no interactions were detected as the traced particles exited the channel without direct contact with the sensing area. Thus, the SHM can be expected to accelerate mass transfer to the biosensor by forcing the target to contact the aptasensor area.

The introduction of active mixing via the spinning microimpeller resulted in crossing particle trajectories and a substantial increase in their velocities (Fig. 4c-iii). The extent, not surprisingly, was found to depend in large part on the impeller spinning rate, a factor that we optimized, as discussed in the Supporting Information, Fig. S4. This increased the target–surface interaction chance by an additional ~5-fold, up to 65%.

**Fig. 5:**
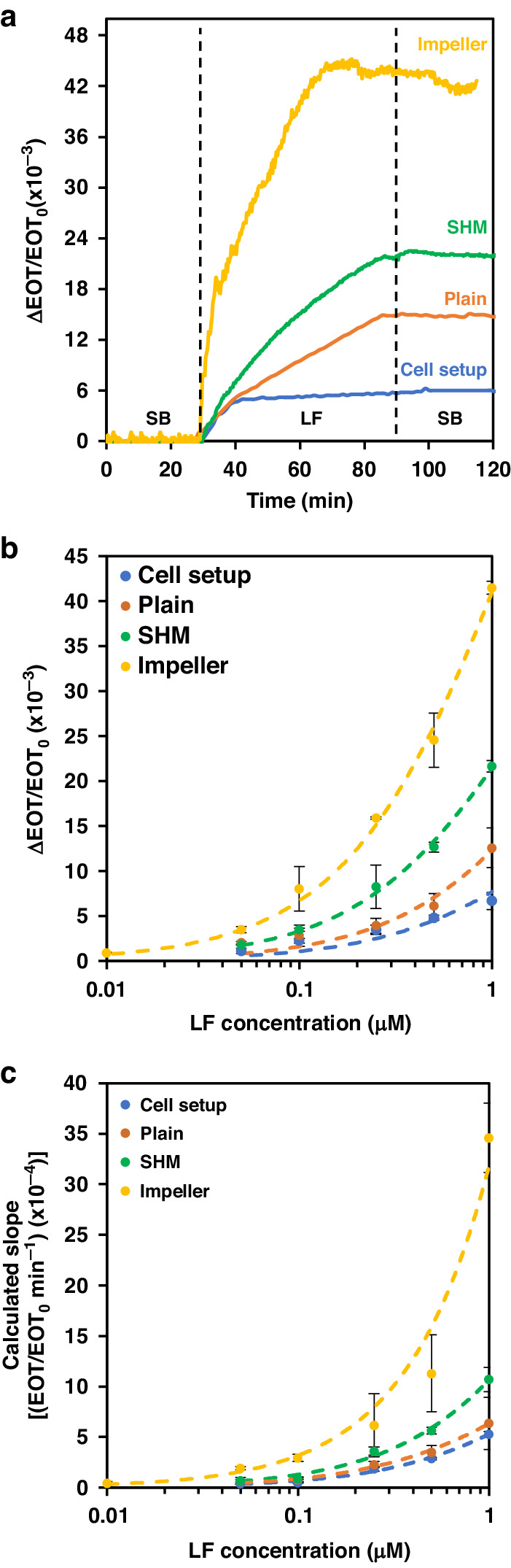
Biosensing performance of integrated aptasensors. **a** Characteristic relative EOT changes as a function of time for 3D-printed microfluidic and non-microfluidic cell setups upon introduction of 1 μM LF. **b** Net relative EOT changes and (**c**) calculated slopes of the integrated aptasensors upon exposure to different LF concentrations

For the biosensing experiments, the aptasensors were integrated within different 3D-printed microfluidic devices, and LF was then introduced at a concentration ranging from 0.01 to 1 µM. Figure 5a presents the characteristic response of the integrated biosensor upon the introduction of 1 μM LF, where a rapid and substantial increase in the EOT signal across the different microfluidic designs was obtained compared to that in the conventional cell setup. Specifically, in the microimpeller system, notable enhancements of ~6-fold and ~7-fold in the net relative EOT signal and the calculated slope, respectively, were observed. This improvement corresponds well with the simulation results, in which the particles-surface interaction increased to 65% (due to the active mixing of the analyte solution), thereby indicating significantly enhanced mass transport of the target to the biosensor surface^40^.

The integration of the SHM was found to induce some enhancement in both the attained slopes and the net EOT changes and resulted in better performance than that observed within the plain microchannel. This result agrees with the simulation results and can be attributed to a greater interaction chance of the target protein within the biosensor. Nevertheless, this improvement is still inferior to that achieved when PDMS-based SHM structures were employed^41,85,89,90^. In the latter case, the SHM grooves were much smaller (with characteristic dimensions of 15–40 µm in height and 40–200 µm in width), and most importantly, groove-to-microchannel height ratios of 20-50% were observed to exhibit optimal mixing efficiency^41,43,91,92^. Owing to the resolution limitations imposed by current 3D printing technology, both the dimensions (groove and microchannel heights of 450 µm and 550 µm, respectively) and the groove-to-microchannel ratio (80%) in our design were by necessity much larger. Indeed, in this case, a substantial volume of fluid was observed to occupy the dead volume within the grooves, as can also be observed in the simulation results (Fig. 4c-ii), leading to a considerably inferior mixing efficiency and an overall reduced biosensing performance^91,93^.

For the plain microchannel, reducing the height of the solution above the porous layer from 1 mm in the cell setup to 550 μm accelerated the target capture rate. An increase in the attained optical signal was observed compared to that in the cell setup, where rapid depletion near the pore entry created a diffusion boundary layer that limited the biosensor sensitivity^4^.

Figure 5b, c compares the binding curves for each of the microfluidic and cell setups, presenting the net relative EOT changes and the infiltration slope vs. the target concentration, respectively. All binding curves displayed a similar trend (best fitted by a sigmoidal curve) across the studied concentration range. Notably, however, the microimpeller system stands out because it enabled detection of LF at a low concentration of 10 nM, which was not detectable by the other setups. Additionally, the integration of the SHM enhanced the performance of the PSi aptasensor compared to that of the plain microchannel and the cell setup, even though the increase in the net ΔEOT remained inferior to that of the microimpeller system. These results agree with our hypothesis and the simulation results, and indicate that employing convection by actively mixing the bulk analyte solution enhances sensitivity by delivering fresh analyte to the biosensor surface, thereby minimizing the effect of the depletion region and reducing the diffusion path length to the pore entry^4,94,95^. Figure 5c depicts the infiltration slopes, which are indicative of the penetration and binding rate of the LF molecules to the tethered aptamers. All setups showed binding curves with similar trends, although yet again the microimpeller outperformed the other setups by exhibiting significantly greater slopes at all concentrations, and thus accelerated infiltration and binding when compared to the passive mixing and nonmixed systems^4,94,95^.

Nevertheless, it should be noted that the impact of convection in porous biosensors seems to be limited; to demonstrate this, we characterized the biosensing performance of an aptasensor with a thicker porous layer of 5 µm. The results presented in Fig. S5 (Supporting Information) reveal that the microimpeller integration yields inferior performance when compared to that of the studied aptasensor of 3 µm—specifically, the calculated slope was 15 times lower for the 5 µm sensor. This finding underscores the critical role of hindered diffusion within the porous layer. Although a thick porous layer offers an increased surface area and more available binding sites, the diffusion and reaction rates are only moderately improved by the acceleration of mass transfer in the bulk solution. These results also agree with our previous simulation results, highlighting the importance of fine-tuning the porous layer thickness^4^ (especially for biosensing interactions with high affinity) where the effects of nanostructure design are more pronounced than those for lower-affinity interactions^4^.

Table 1 summarizes the analytical performance of these microfluidic-integrated systems and reveals that microimpeller integration induced a >1 order of magnitude enhancement in the LOD of the biosensor compared with that of the conventional cell setup, which is relevant for LF detection in real clinical samples. These results demonstrate the significant role of mass transfer acceleration strategies in PSi-based biosensors since target flow induces a greater signal across all systems compared to that for the cell system.

**Table 1:** Analytical performance of the 3D-printed microfluidic-integrated aptasensor compared to that of a non-microfluidic cell setup

	3D-printed microfluidic setup			Cell setup
	Plain microchannel	SHM-structured microchannel	Impeller-integrated microchannel	
SNR^a^	2.7 ± 0.3	1.5 ± 0.6	4.8 ± 0.6	2.3 ± 0.7
LOD (nM)	35	20	3	50
LOQ (nM)	110	50	8	140
%RSD	8–24	3–27	1–25	4–25
*K*_D_ (µM)	2 ± 1	1.6 ± 0.6	1.3 ± 0.6	2 ± 1

^a^For a 50 nM LF concentration

The selectivity of the integrated biosensors upon exposure to the nontarget protein AGR2 was studied, and the results are presented in Fig. S6a (Supporting Information). The resulting EOT signal was minimal for the different microfluidics. Interestingly, however, both the SHM and impeller configurations were found to improve the selectivity of the biosensor. This is demonstrated by the high values of the calculated ratio between the attained signals for LF and AGR2 (Fig. S6b, Supporting Information), possibly owing to an improved immobilization of the passivating PEG molecules which was carried out under convection in the case of the integrated aptasensors.

## Conclusions

In conclusion, in this work we report the design and construction of a 3D-printed microfluidic-integrated PSi aptasensor aimed at detecting the biomarker LF, and evaluate different approaches for extending the sensitivity of the system toward clinically relevant concentrations.

We demonstrated the crucial impact of various structural properties of the PSi transducer on the biosensor sensitivity. This stems from mass transfer limitations in these structures due to the bulk diffusion of the target toward the surface of the biosensor, the hindered diffusion within the porous layer, and the target-capture probe reaction. Our results demonstrate that the aptamer affinity, surface density, pore diameter, and porous layer thickness are all factors that have a substantial effect on the sensitivity, resulting in a >1 order of magnitude lower LOD compared to previously studied PSi-based aptasensors.

In addition, the resulting PSi aptasensor was incorporated into 3D-printed microfluidic systems with two different designs: SHM-structured and impeller-integrated microchannels. This integration aimed to enhance convective flow within the system and increase the chance of surface interactions of the target analyte. The aptasensor sensitivity (with an LOD of 50 nM) was improved by integrating it into the different microfluidic setups; specifically, within the SHM-structured microchannel, the LOD decreased by ~2.5-fold compared to that in the cell setup and by ~2-fold compared to that in the plain microchannel. 3D-printed SHM structures with reduced dimensions are expected to further improve the biosensing performance. However, owing to the currently limited resolution of 3D printers, such structures are challenging to produce. Still, 3D printing technology is rapidly advancing at this point, the resolution continues to improve, and printers with capabilities in the lower micrometer range are already available. The impeller-integrated microchannel produced a >1 order of magnitude lower LOD of 3 nM, which is relevant for LF detection in real clinical samples. The aptasensor exhibited lower selectivity and sensitivity within the complex GI fluid; however, our future work will be directed toward exploring new surface passivation strategies with the aim of enhancing the sensor efficacy in complex media. Furthermore, future investigations will extend to the examination of signal amplification methodologies, such as the deployment of secondary capture probes.

### Supplementary information

Supplementary information
