# Supplementary material for: Enhancing the performance of porous silicon biosensors: the interplay of nanostructure design and microfluidic integration
Source: Microsyst Nanoeng. 2024 Jul 17;10:100. doi: 10.1038/s41378-024-00738-w (PMC11252414; doi:10.1038/s41378-024-00738-w)
Supplement: Supplementary file 1 — Supplementary information [file 41378_2024_738_MOESM1_ESM.docx]

# Supporting Information

## PSi Aptasensor Construction

**Table S1:** Etching conditions and characterization results obtained by SLIM and SEM

| **Etching conditions** | | | **HRSEM** | | **SLIM** | | **Fringes** |
| --- | --- | --- | --- | --- | --- | --- | --- |
| **Electrolyte (HF:EtOH)** | **Etching time (s)** | **Current density (mA cm^-2^)** | **Pore diameter (nm)** | **Thickness (nm)** | **Open porosity (%)** | **Thickness (nm)** |  |
| 1:1 | 120 | 75 | 80 ± 10 | 4750±10 | 84 ± 5 | 4900 ± 200 |  |
| 1:1 | 70 | 75 | 80 ± 10 | 2800 ± 200 | 89 ± 2 | 3000 ± 100 |  |
| 3:1 | 30 | 375 | 50 ± 10 | 4550 ± 90 | 74 ± 4 | 4720 ± 90 |  |
| 3:1 | 12 | 375 | 50 ± 10 | 2410 ± 40 | 79 ± 5 | 2730 ± 90 |  |
| 3:1 | 5 | 375 | n/A | n/A | n/A | n/A |  |

*The PSi layer fabricated by electrochemical etching at 375 mA cm^-2^ for 5 s could not be analyzed by SLIM.

## Aptasensor Selectivity and Sensitivity


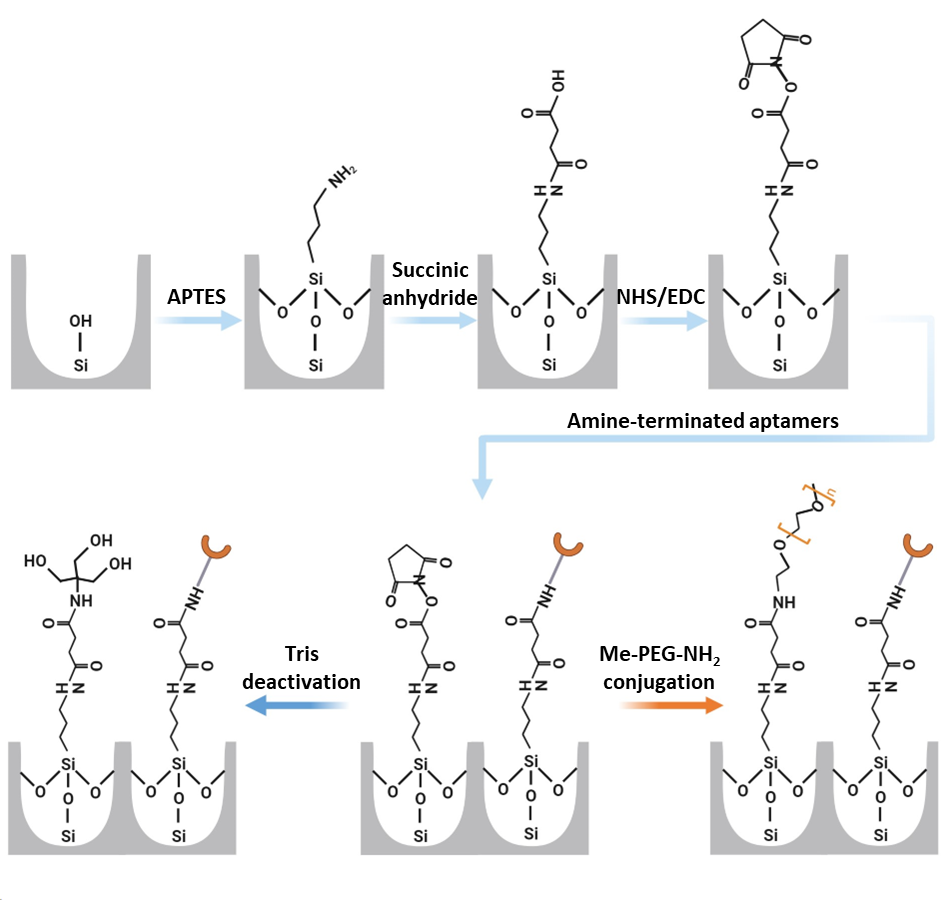


**Figure S1:** Schematic illustration of oxidized PSi functionalization with aptamers: silanization of the oxidized PSi surface with APTES to create an amine-modified surface; carboxylation with succinic anhydride; NHS/EDC activation; and covalent binding of the amino-modified aptamer, with different passivation strategies.


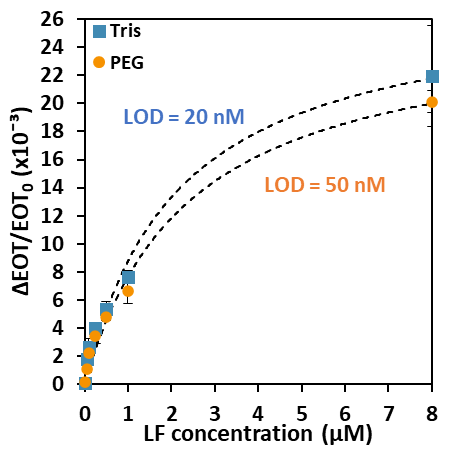


**Figure S2:** Characterization of the biosensing performance of the aptasensor in selection buffer.

## Mass Transfer Acceleration


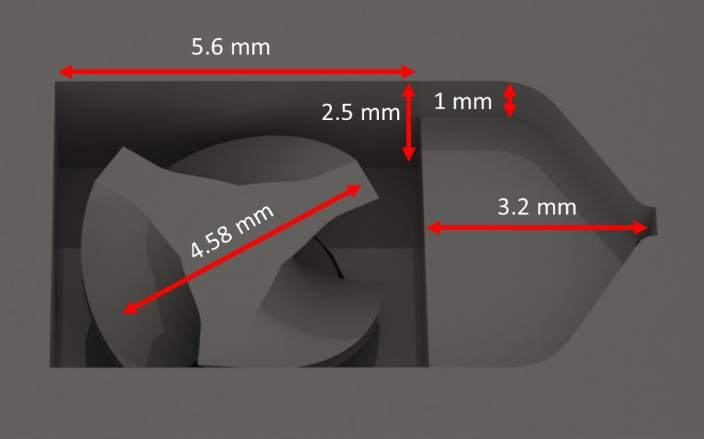


**Figure S3:** Dimensions of the microimpeller system.

The performance of the biosensor with the impeller-integrated microchannel was studied for different stirring rates, and an optimal signal was observed at 970 rpm (see Figure S4). A further increase in the stirring rate decreased the EOT signal, which can be attributed to protein denaturation.


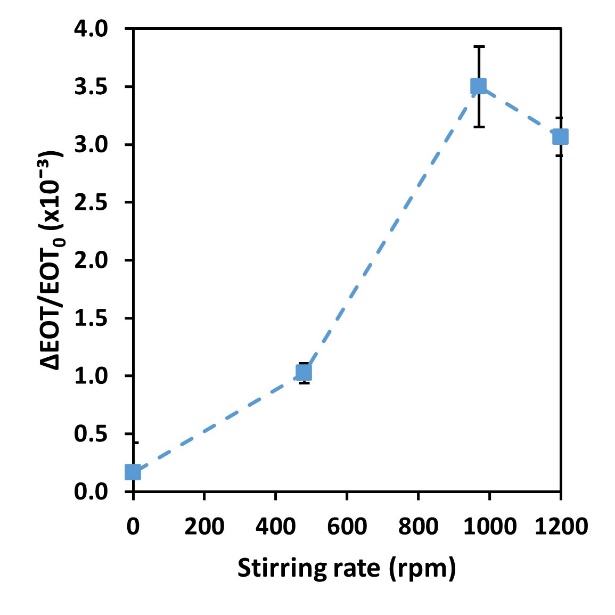


**Figure S4:** Net relative EOT changes of the biosensor integrated in an impeller-integrated 3D-printed microchannel system upon exposure to 0.05 µM LF with different stirring rates of 0, 480, 970 and 1200 rpm.


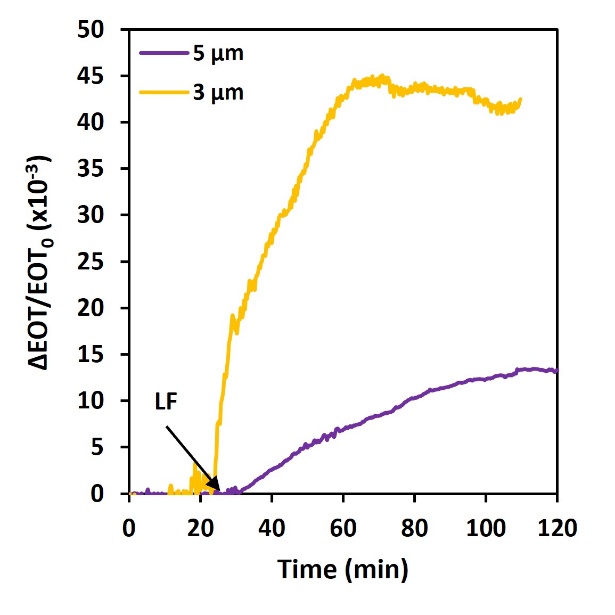


**Figure S5:** Characteristic relative EOT changes as a function of time for PSi aptasensors with different porous layer thicknesses integrated within the microimpeller system.


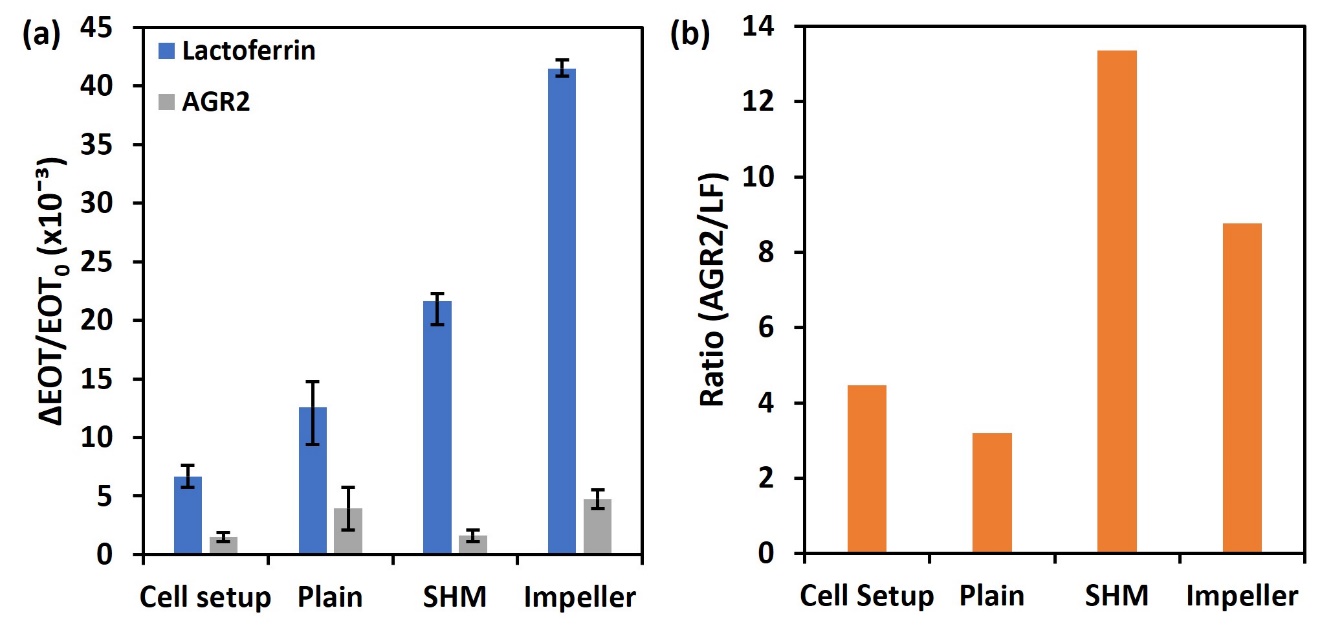


**Figure S6:** (a) Net relative EOT changes of the biosensor integrated in the different 3D-printed microfluidic systems upon exposure to LF and AGR2 at a similar concentration of 90 μg mL^−1^, which is equivalent to 1 µM for LF. (b) Ratio between the signal intensity for LF and that for the nontarget protein AGR2.
